# Ubiquitination-related Gene UBTD1 Mediates Poor Prognosis of Colorectal Cancer and Affects Colorectal Cancer Cell Proliferation and Ferroptosis

**DOI:** 10.2174/0115748928323408241002131753

**Published:** 2025-01-15

**Authors:** Yuzhao Jin, Luyu Liao, Lingjiao Guo, Qianping Chen, Bufu Tang, Jin Jiang, Ji Zhu, Minghua Bai

**Affiliations:** 1 Postgraduate training base Alliance of Wenzhou Medical University (Zhejiang Cancer Hospital), Hangzhou, Zhejiang, 310022, China;; 2 Hangzhou Institute of Medicine (HIM), Chinese Academy of Sciences Hangzhou, 310000, China;; 3 Department of Abdominal Radiotherapy, Zhejiang Cancer Hospital, Hangzhou, 310000, China;; 4 Yuhuan Second People's Hospital, Health Community Group of Yuhuan Second People's Hospital, Taizhou, 317605, China;; 5 Department of Radiotherapy, Zhongshan Hospital, Shanghai, 200032, China;; 6 Department of Radiotherapy, First Hospital of Jiaxing, Jiaxing, 31400, China

**Keywords:** CRC, immune escape, ferroptosis, ubiquitination, UBTD1, prognosis, cell proliferation

## Abstract

**Background:**

Colorectal cancer (CRC) is the third most common cancer worldwide, and its occurrence and progression are often regulated by genetic and hereditary factors. Ubiquitination and the associated ubiquitin-binding enzymes and ligases regulate the tumor microenvironment and antitumor immunity to mediate tumor pathogenesis and progression. In this study, we examined the molecular characteristics and immunomodulatory effects of ubiquitination-associated genes that mediate CRC prognosis.

**Methods:**

The ubiquitination-related gene ubiquitin domain-containing protein 1 (UBTD1) was identified using bioinformatics and single-cell analyses. Subsequently, the ability of UBTD1 to predict CRC prognosis and immune checkpoint correlation was analyzed, the potential drug telatinib targeting UBTD1 was explored, and the correlation between UBTD1 and ferroptosis was analyzed. The role of UBTD1 in CRC and ferroptosis was verified using immunohistochemistry, gene knockout, western blot, cell cloning, and immunofluorescence.

**Results:**

UBTD1 was identified as a significant prognostic and predictive gene for CRC and was involved in regulating immune checkpoint levels and immune cell function of CRC patients with CRC. High UBTD1 expression was found to enhance the presence of immune checkpoints that induce immune escape and inhibit ferroptosis onset. Telatinib may be a potential therapeutic drug targeting UBTD1.

**Discussion:**

This study first identifies UBTD1 as an independent prognostic marker for CRC, with high expression correlating with poor patient survival. Mechanistically, UBTD1 promotes tumor immune escape by upregulating key immune checkpoints and inhibits ferroptosis via regulating GPX4 and lipid peroxidation, thereby facilitating CRC progression. Telatinib shows strong binding affinity with UBTD1 and inhibits CRC cell viability, offering a potential targeted therapy for high UBTD1-expressing CRC. These findings link ubiquitination, immune escape, and ferroptosis in CRC, providing a novel molecular target for precision treatment.

**Conclusion:**

Our study demonstrated that UBTD1 is a prognostic marker for CRC in the regulation of ubiquitination and the tumor immune microenvironment and may serve as a modulator of ferroptosis.

## 1 INTRODUCTION

Colon cancer is the third most common cancer worldwide (approximately 10.2% of all cancer cases) and the second most common cause of cancer-related deaths (approximately 9.2% of all cancer-related deaths) [1]. Surgery combined with chemoradiotherapy remains the primary treatment for early-stage tumors [2]. However, approximately 20% of patients have already metastasized at the first diagnosis, and 25-30% of advanced patients relapse within five years after curative surgery [3]. Over the past decade, neoadjuvant chemoradiotherapy, molecularly targeted drugs, and immune checkpoint inhibitors have demonstrated substantial benefits in patients with advanced or surgery-intolerant CRC [4, 5]. Furthermore, the discovery of dMMR/MSI-H and a high mutation burden (TMB) underpin evidence-based medicine for molecular classification in conjunction with immunotherapy [6, 7]. Cancer pathogenesis has been suggested to be regulated by genetic and hereditary factors. Therefore, it is expected that identifying novel biomarkers and potential targets will facilitate the advanced prediction and efficacy assessment of CRC.

Ubiquitination is a multi-step enzymatic process that modifies target proteins by binding to ubiquitin (a class of low-molecular-weight proteins) molecules [8]. In particular, the ubiquitin system is activated by activating enzyme E1 in an ATP-dependent manner, and ubiquitin is then transferred from E1 to conjugating enzyme E2. Ubiquitin is then specifically conjugated to substrates by the ligase enzyme E3. The ubiquitin-proteasome system (UPS) degrades proteins through proteasomes and regulates a wide range of cellular processes. Moreover, ubiquitination is a reversible process whereby deubiquitinating enzymes (DUB) specifically reverse ubiquitination degradation to stabilize substrate protein expression [9, 10].

Ubiquitination plays a crucial role in cancer development. Genomic instability caused by deregulation of the UPS is a hallmark of cancer [11]. Aberrations in the UPS in the regulation of inflammatory responses or DNA repair processes also lead to improper or inadequate assembly of protein complexes, ultimately leading to derangements in cellular metabolism and tumorigenesis. Ubiquitination is also key to the dynamic regulation of programmed cell death [12]. Numerous studies have reported that dysfunction or aberrant expression of ubiquitination and its associated ubiquitin ligases, which are critical regulators of programmed cell death, modify the prognosis of patients with tumors [13]. Many ubiquitin ligases and DUBs have been identified as regulators of antitumor and tumor-mediated immunosuppression in cancers [11, 14, 15]. Several proteins involved in ubiquitination have been identified as potential targets for cancer treatment; however, their molecular mechanisms and pathways remain unclear.

Ubiquitin domain-containing protein 1 (UBTD1) encodes a protein consisting of 227 amino acids with a molecular weight of 26 kDa and is thought to regulate E2 ubiquitin-conjugating enzymes belonging to the UBE2D family [16]. It has been reported that UBTD1 mediates the ubiquitination of YAP, thereby affecting YAP downstream signaling and MDM2 ubiquitination and increasing the stability of P53, which may affect tumorigenesis [17-19]. Further research is necessary to explore the role of UBTD1 in cancer development, its prognostic potential, and its effects on immune-related regulation.

Ferroptosis, a newly characterized form of iron-dependent cell death driven by lipid peroxidation, has emerged as a critical process in the regulation of cancer cell survival [20, 21]. Targeting ferroptosis as a potential therapeutic strategy has demonstrated potent anticancer activity in a variety of cancers [22-24]. Understanding how UBTD1 influences ferroptosis could provide valuable insights into overcoming resistance to current therapies.

Given the critical roles of ubiquitination and ferroptosis in cancer biology and the potential of UBTD1 as a novel prognostic marker and therapeutic target, this study aims to elucidate the molecular mechanisms by which UBTD1 influences CRC progression and immune evasion, thereby contributing to the development of more effective treatment strategies for CRC patients. In this study, we used bioinformatic analysis of CRC data to investigate the involvement of ubiquitination-related prognostic genes. We identified UBTD1 as a regulatory factor closely related to the immunogenic profile and prognosis of CRC patients. We also evaluated the relationship between UBTD1 and immune cell infiltration into the tumor microenvironment, immune checkpoint levels, and ferroptosis sensitivity. Therefore, our results contribute to the assessment of CRC prognosis and the establishment of individualized therapeutic regimens.

## 2 MATERIALS AND METHODS

### 2.1 Collection of Ubiquitination-related Genes

One thousand three hundred sixty-four ubiquitination-related genes were collected from the iUUCD 2.0 database (http://iuucd.biocuckoo.org/) [25], while 478 ubiquitination-related genes were obtained from various works of literature. After eliminating duplicate genes, we recorded ubiquitination-related genes in **Supplementary Table S1**.

### 2.2 Acquisition of Datasets

Transcriptome data of 44 normal colorectal tissues and 565 colorectal cancer tissues, together with their corresponding clinical information, were obtained from TCGA-COAD&READ cohorts in the Cancer Genome Atlas (TCGA) database. The mRNA expression profiles and clinical information of CRC were downloaded from the GSE39528 database (https://www.ncbi.nlm.nih.gov/geo/query/acc.cgi?acc=GSE39582) and the GSE87211 database (https://www.ncbi.nlm.nih.gov/geo/query/acc.cgi?acc=GSE87211).

### 2.3 Identification of Differential Prognostic Ubiquitination-related Genes (DPURGs)

The mRNA sequences with 1458 ubiquitination-related genes were matched and analyzed by the “edgeR” package. Log2 fold change (FC) > 1.0 and adjusted *P* value<0.05 were considered to select differentially expressed genes (DEGs). Then, univariate Cox regression through the “Survival” R package was used to identify genes linked with the overall survival (OS) rate in patients with colorectal cancer. Significant genes associated with OS were determined when the *P*-value was less than 0.05.

### 2.4 Gene set Variation Analysis (GSVA)

Gene set variation analysis (GSVA) is a particular gene set enrichment method that enables pathway-centric analyses of molecular data from genes to gene sets. The gene sets of cancer hallmarks were obtained from the Molecular Signatures Database (https://www.gsea-msigdb.org/gsea/msi gdb/). TCGA gene datasets were then used to obtain the score of each sample in each hallmark pathway by the “GSVA” R package. With the GSVA score, the correlation between DPURGs and hallmarks was explored. Similarly, using GSVA scores, the correlation of UBTD1 with the mode of tumor death was investigated, and the association of UBTD1 with ferroptosis-related pathways was further analyzed.

### 2.5 Immune Landscape and Correlation with DPURGs

The TIDE score, dysfunction score, MSI score, and exclusion score were obtained from the TIDE database [26] (http://tide.dfci.harvard.edu/). TIDE Database systematically identifies genes in the Cancer Genome Database to screen for genes that affect cytotoxic T cell function in terms of patient survival outcomes, testing the interaction between candidate genes and cytotoxic T cell function using the Cox-PH model, where the z-score for each gene is obtained by dividing the interaction coefficient d by its standard error. Each score can be calculated by inputting the gene matrix. TIDE score prediction score can be interpreted as a z-score, and a higher value indicates a higher potential for tumor immune evasion. Dysfunction scores can predicate the potential of T cell dysfunction in the tumor. MSI score is trained from the TCGA database through Ridge Regression and used to predicate MSI. The exclusion score can predicate the T-cell exclusion potential in the tumor. These are composite scores for tumor immune dysfunction and immune escape, which can predict the efficacy of immune checkpoint inhibitors.

Moreover, the gene expression of immune checkpoints in patients with CRC was collected from the TCGA database. Using the Cell-type Identification by Estimating Relative Subsets of RNA Transcripts (CIBERSORT) analysis, we converted transcriptome data to the abundance of immune cells and stromal cells in colorectal cancer by using a deconvolution technique known as CIBERSORT [27]. The required reference 22 immune-cell dataset was downloaded from CIBERSORTX (https://cibersortx.stanford.edu/). The R package was “CIBERSORT.” Correlation heatmaps were used to exhibit correlation and statistical significance with DPURGs, and the method was “Spearman.”

To explore the effects of UBTD1 expression on immune cells, immune functions, and the potential efficacy of immune checkpoint inhibitors (ICIs), we determined the immune-related differences in two groups using the methods described above and the relationship between UBTD1 and classic checkpoints like PD-1 and CTLA4.

### 2.6 Single-cell Analysis of Cell Positions of DPURGs

The transcriptomes of 33,482 single human CRC cell data were obtained from the GSE188711 database. The R package “Seurat” was used for Principal Component Analysis (PCA), and then the t-distributed Stochastic Neighbor Embedding (tSNE) was run. In this single-cell sequencing object screening process, single genes were expressed in at least 10 cells, single cells expressed at least 200 genes, and high-quality cells were retained according to nFeature_RNA>500, nCount_RNA>1000, nCount_RNA <20000, and percent. mt<10. After screening and cell cycle heterogeneity correction, 18078 cells were left. The PCA number was 18, and cells were divided into 15 groups with 10 classes. The cell population was annotated based on the R package “Single R” and feature markers. Then, the relative content and location of DPURGs were visualized in feature plots.

### 2.7 Exploration of Potential Drug and Semiflexible Docking with UBTD1

RNA expression data for the drug were obtained from the CellMiner database [28] (https://discover.nci.nih.gov/cellminer/home.do). Analysis of DPURG RNA expression was based on the mean Z score of activity (DTP NCI-60). The drugs with the highest correlation coefficients, which indicated the most sensitive, were selected, and tried to connect in semi-tandem by AutoDock Vina software. Open Babel was used to adjust the 3D structure from the PubChem database (https://pubchem.ncbi.nlm.nih.gov/). In addition, the 3D design of UBTD1 was obtained from the protein database PDB (https://www.rcsb.org).

The “OncoPredict” R package was used to predict drug sensitivity scores when provided with bulk RNAseq gene expression data [29]. The GDSC database [30] (https://www.cancerrxgene.org/) was utilized as the practicing set, and the TCGA database as the validation set. The drug score differences between the two groups were obtained to predict sensitive drugs against UBTD1.

### 2.8 Assessment of the Prognostic Significance of Different Features and Establishing and Evaluating the Nomogram

To evaluate the clinical prognostic significance of each clinical feature and expression of UBTD1, COX regression analysis was performed. Subsequently, the “RMS” package was applied to develop a nomogram that combines different clinical features and UBTD1 expression for quantitative prognosis prediction in patients with CRC. Based on a calibration curve, the calibration of the nomogram was evaluated to determine if the prediction of survival rates matched the actual survival rates. Furthermore, survival benefits were assessed *via* Decision Constructed Curve Analysis (DCA).

### 2.9 Survival Analysis

The effect of alternations in UBTD1 expression on OS was assessed by analyzing the Kaplan-Meier survival curve (KM). ROC curves were calculated by “survival ROC” to predict system survival for 1, 3, and 5 years. The area under the ROC curve (AUC) was calculated to predict prognosis.

### 2.10 Gene set Enrichment Analysis (GSEA)

The transcriptome expression following CRISPR knockdown of UBTD1 in breast cancer cells was obtained from the GSE187008 dataset (https://www.ncbi.nlm.nih.gov/geo/query/acc.cgi?acc=GSE187008), and “limma” R package was applied to find DEGs. DEGs in the high and low UBTD1 expression groups were also computed. We then performed enrichment analyses of DEGs using KEGG, GO, and HALLMARKS (https://www.gsea-msigdb.org/gsea/index.jsp).

### 2.11 Correlation between Immune Microenvironment and UBTD1 Expression

In order to assess the proportion of stromal cells and immune cells within the immune microenvironment, the “estimate” R package was used in two groups of CRC patients to compute the immune scores, the stromal scores, and tumor purity (https://sourceforge.net/projects/estimateproject/).

### 2.12 Statistical Analysis

All data were computed and visualized by R software (4.2.1). A list of the required R packages can be found above. GraphPad Prism 9.5 was used to plot histograms. When the *P*-value is less than 0.05, it is considered statistically significant.

### 2.13 Cell Viability Assay

CCK8 was used to detect the toxicity of Telatinib on CRC cells. Briefly, DLD1 cells (3x10^3^ cells/well) were inoculated in 96-well plates, and the next day, a gradient dilution of Telatinib was added and incubated for 48 h. Two days later, 10 µl of CCK8 was added to each well and incubated for 60 min at 37°C in a thermostat, and the absorbance at 450 nm was measured using an enzyme marker. The data were recorded and analyzed for IC50.

### 2.14 Immunohistochemistry

We obtained pairs of samples from colorectal cancer and adjacent tissues from Zhejiang Cancer Hospital, first fixed the tissue with formalin for 24 hours, then dehydrated it with 75% ethanol, embedded it in paraffin, and cut it into 4 μm thick paraffin sections. We used anti-UBTD1 antibodies (UBTD1 Antibody, SAB#35126, 1:100) stained overnight; then, the sections were washed and stained with a suitable secondary antibody. All sections were stained under an Olympus microscope.

To quantitatively analyze immunohistochemistry results, the plugin “IHC profiler” of image J was used to calculate the IHC scores.

### 2.15 Cell Transfection

To knock down UBTD1, SiRNA targeting UBTD1 (Si-UBTD1#1:5' -GGAGACCAAGATCCAGAAA-3'; Si-UBTD1#2:5' -GAGCGGCTTAAGTGGAAGA-3') was purchased from Ribobio Corporation (Guangzhou, China). According to the instructions, buffer, SiRNA, reagent, and culture medium were added to prepare a SiRNA mixture with a final concentration of 50 nmol and incubated at room temperature for 15 minutes. The transfection mixture was added dropwise to the cells, and the plates were gently rocked to ensure even distribution. Cells were incubated at 37°C with 5% CO_2_ for 6 hours, after which the medium was replaced with a complete medium containing 10% FBS and no antibiotics. The cells were further incubated for 72 hours before being harvested for subsequent experiments or protein extraction.

### 2.16 *In vitro* Proliferation Assay

DLD1 cells of SiNC and SiUBTD1 were separately spread in 6-well plates; each well was planted with 150, placed in a 37°C thermostat, and cultured with complete medium for 12 days. After 12 days, the medium was aspirated, the slides were washed with PBS, fixed with 4% paraformaldehyde, and then stained with 0.5% crystal violet dye for 30 minutes, and finally, the dye was washed off and air-dried for counting.

Colorectal tumor tissue specimens were digested into individual cells and placed separately in organoid basal medium. The transfection method was the same as above and was observed for 9 consecutive days and photographed and recorded.

### 2.17 Western Blot Analysis

RIPA lysate was used to extract the transfected proteins. Lysate proteins were subsequently separated by electrophoresis and transferred to nitrocellulose membranes, incubated with primary antibodies (UBTD1 Antibody, SAB#35126; GPX4 Antibody, abcam#ab120566; SLC7A11 Antibody, CST#12691S; Tubulin Antibody, Sigma#T9026), overnight at 4°C. The following day, the membrane was washed with TBST for 3x10 mins, incubated with the corresponding secondary antibody for 90 minutes, and then washed with the membrane for 3x10 mins. Finally, the bands were developed with an ECL developer.

### 2.18 Lipid Peroxide Determination

Liperfluo-Cellular Lipid Peroxide Assay Kit (#L248) was purchased from DOJINDO Corporation (Japan). Liperfluo was dissolved in 60 µl DMSO, heated in 40°C water for 30 min to aid dissolution, and after complete dissolution, 5960 µl of serum-free DMEM was added to configure Liperfluo at a concentration of 10 µM.

The 24-well plates with 10,000 cells/well were laid out in advance and divided into 2 groups, one for DLD1 and the other for DLD1-SiUBTD1. Then, 50 µM of Erastin was added to the DLD1 group; 50 µM of Erastin, 5 µM of Fer-1, and 50 µM of Erastin + 5 µM of Fer-1 were added to another group, incubated at 37°C for 24 h, washed twice with PBS the next day, then 250 µl Liperfluo was added and incubated in a constant temperature incubator for 30 min, washed twice with PBS, incubated for 10 min with 1 µg/ml of DAPI, and washed twice with PBS. In the end, 250 µl PBS was added to each well. A high-content imager was used to capture the 40x staining situation, and lipid peroxidation was compared by detecting fluorescence levels.

## 3 RESULTS

### 3.1 Acquisition of Differentially and Prognostically Expressed Genes in CRC

**Fig. (1):**
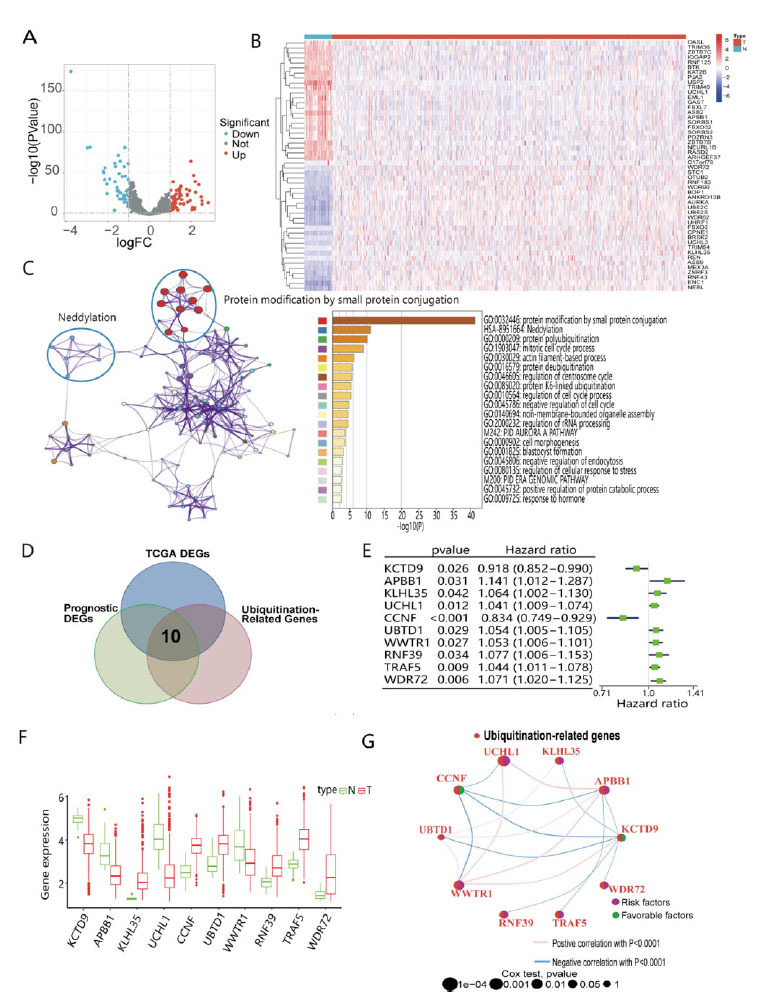
Identification of the expression, biological function, prognostic value, and correlation of DPURGs in CRC. (**A**) The volcano plot shows 114 DEGs related to ubiquitination in the TCGA cohort. (**B**) TCGA cohort had the top 50 DEGs associated with ubiquitination. (**C**) GO enrichment analysis of the DEGs. Proteins with the same function are shown in the same color. Function descriptions are sorted by *P*-values and correspond to colors. (**D**) Ten independent prognostic genes are shown in the Venn diagram. (**E**) Forest plot of DPURGs risk ratio. (**F**) The expression of 10 DPURGs between normal and CRC tissues from the TCGA cohort. (**G**) Correlations between DPURGs in TCGA cohort. Positive correlations are represented by pink lines, and negative correlations are represented by blue lines (*P* <0.0001).

As the molecular mechanisms of ubiquitination-related regulatory genes in CRC remain unclear, we explored the clinical significance and immune landscape of differentially prognostic ubiquitination-related genes (DPURGs). First, we searched databases and various studies on ubiquitination-associated genes and recorded them, as illustrated in **Supplementary Table S1** [25, 31-33]. Among them, 114 genes were differentially expressed in colorectal and paracancerous tissues (**Supplementary Table S2**) and are presented as volcano and heat maps (Figs. **1A** and **B**). To explore the biological functions of these differentially expressed genes (DEGs), we performed a Gene Ontology (GO) analysis. Small protein conjugation and neddylation were the most enriched biological functions, further illustrating the reliability of the ubiquitination database (Fig. **1C**).

Univariate Cox regression analysis was conducted to investigate the relationship between 114 DEGs and overall survival (OS). Fig. (**1D**) shows that only ten genes could serve as DPURGs that regulate the prognosis of CRC patients. We performed survival analysis on the genes and plotted the KM curves, as shown in Figs. (**S1A**-**J**). Fig. (**1E**) shows the hazard ratio of the 10 DPURGS. Compared to normal tissues, the expression of six genes was upregulated, and that of four genes was downregulated in CRC tissues (Fig. **1F**). We then performed a correlation analysis of these DPURGs and found that only CCNF and KCTD9 were favorable factors, whereas the remaining eight genes were risk factors (Fig. **1G**). We calculated the correlation coefficients between all the DPURGs. Our results revealed that the correlation between the expression levels of the six groups of genes was greater than 0.3, suggesting a synergistic effect (Fig. **S1K**).

### 3.2 Single-cell Analysis of DPURGs from Different Colorectal Cancer Cells

To investigate the relationship between DPURGs and CRC cells, we analyzed the expression of DPURGs in CRC cells using a CRC single-cell library. We obtained transcriptomic data from the GSE188711 database of over 30,000 human CRC monocytes. The quality control and filtering processes were performed as described in the Methods section. Subsequently, we screened the top 2000 highly heterozygous genes, as shown in Fig. (**S2A**). Principal component analysis of these genes was performed using a scree plot, and the PC num was determined to be 18 (Fig. **S2B**).

Fifteen cell populations were annotated in combination with their characteristic markers, as shown in Fig. (**S2C**). Fig. (**S2D**) shows that DPURGs are commonly expressed in T lymphocytes, B lymphocytes, monocytes, macrophages, epithelial cells, smooth muscle cells, CMP, fibroblasts, endothelial cells, neutrophils, and DC. Notably, KCTD9, APBB1, and TRAF5 were evident in immune cells, such as T and B lymphocytes. UBTD1 is highly expressed in macrophages, epithelial cells, and smooth muscle cells and is partially expressed in DCs (Fig. **S3A**). These results implied that DPURGs are locally abundant on the surface of immune cells and potentially regulate immune system activation and transmission.

We also investigated the correlation between UBTD1 expression and macrophages. We divided the macrophages into two groups based on UBTD1 expression and compared differences in gene expression and pathways. Fig. (**S3B**) shows that UBTD1 may affect macrophages by affecting CD74, the receptor for the macrophage migration inhibitory factor. CHI3L1 may be associated with macrophage differentiation. In addition, UBTD1 may influence the immunotherapeutic effects of IL1RN, FCGR3A, CD74, LILRB5, and SPP1. Kyoto Encyclopedia of Genes and Genomes (KEGG) analysis of the DEGs revealed that macrophages with differential UBTD1 expression were mainly involved in the reactive oxygen species oxidative phosphorylation, mTOR, and xenobiotic metabolism pathways (Fig. **S3C**).

### 3.3 Relationships between DPURGs, Immune Checkpoints, and Escape Ability

**Fig. (2):**
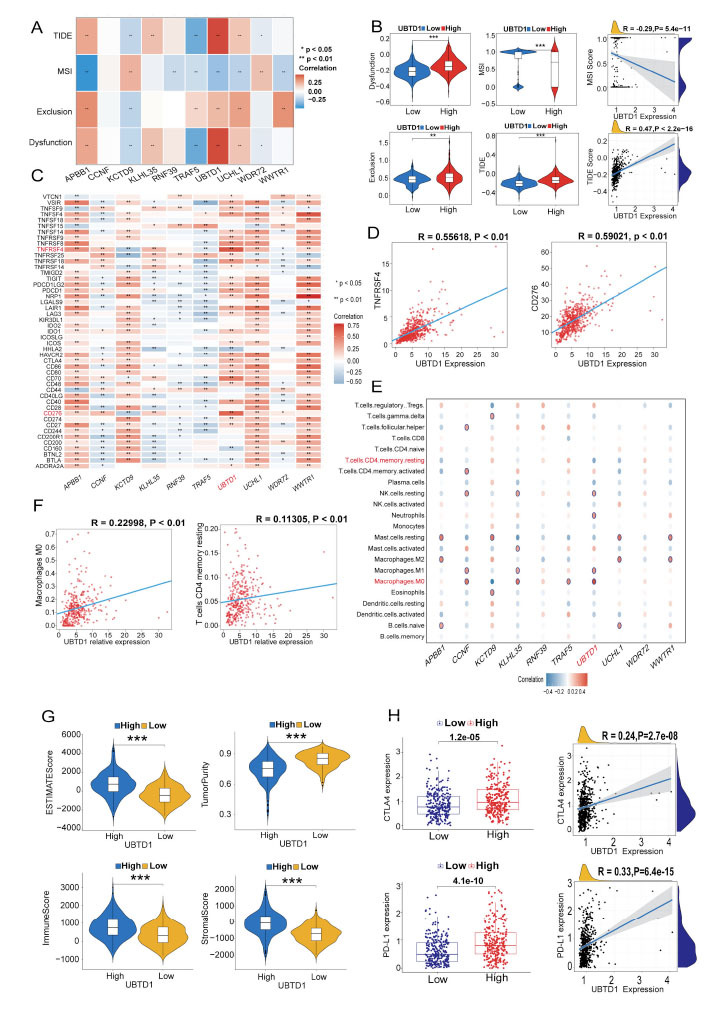
Correlation between DPURGs and immune characteristics. (**A**) Correlation of TIDE, MSI, exclusion, and dysfunction scores with DPURGs scores. (**B**) Distribution of TIDE, MSI, exclusion, and dysfunction scores based on UBTD1 expression. (**C**) Correlation between DPURG expression and immune checkpoints. (**D**) Scatter plots showing the relative expression of TNFRSF4, CD276, and UBTD1. (**E**) Infiltration levels of 22 immune cells correlated with DPURG expression. (**F**) Scatter plots showing the correlation between Macrophages M0 and CD4+ memory T cell and UBTD1 relative expression. (**G**) Violin plots showing the distribution of ESTIMATE scores, tumor purity, immune scores, and stromal scores in high- and low-UBTD1 groups. (**H**) CTLA4 and PD-L1 are expressed in the high- and low-expression UBTD1 tumors. Correlation of UBTD1 expression with the immune checkpoints CTLA4 and PD-L1.

The TIDE, MSI, exclusion, and dysfunction scores of the TCGA cohort were calculated to further investigate the correlation between genes and immunity. We calculated the correlation and *P*-values using the Spearman method (Fig. **2A**). Generally, a higher TIDE score indicates greater potential for immune escape from the body. Genes with high tumor immune dysfunction and rejection (TIDE) scores can serve as biomarkers and modulators of immune checkpoint inhibitory therapy resistance. The heatmap revealed that among the DPURGs, UBTD1 achieved the highest TIDE and dysfunction scores, suggesting a role for UBTD1 in immune escape.

In the following analysis, we compared the TIDE, MSI, exclusion, and dysfunction scores between the two groups and found a significant difference between the two groups. Furthermore, the positive correlation between UBTD1 expression and TIDE score (R = 0.47, *P* = 2.2 E-16), as determined by the correlation analysis, indicated that UBTD1 plays a role in aiding tumors to evade the immune system (Fig. **2B**).

We also conducted an analysis of immune cell infiltration and immune checkpoints for UBTD1. The results revealed that immune cell infiltration and immune checkpoints were significantly different in the presence of DPURGs (Figs. **2C** and **E**). In particular, UBTD1 expression was positively correlated with the immune checkpoints TNFRSF4 (R = 0.55618), CD276 (R = 0.59021), immune cell Macrophage M0 (R = 0.22998), and CD4+ memory T cells (R = 0.11305) (Figs. **2D** and **F**). TNFRSF4 facilitates the expression of the apoptosis suppressors BCL2 and BCL2L1/BCL2-XL, thereby inhibiting apoptosis [34]. CD276 is highly expressed in cancer stem cells and is used to evade immune surveillance during tumorigenesis, cancer progression, and metastasis [35]. Consequently, UBTD1 may be a potential therapeutic target as a resistance modulator for immune checkpoint inhibition therapy.

To further investigate the potential mechanism of UBTD1-induced immune escape, we performed immune correlation analysis for the high and low UBTD1 expression groups. Furthermore, the immune cell score, stromal cell score, and tumor purity analysis of CRC tissues showed that the highly expressed UBTD1 tumor tissues had a greater number of immune and stromal cells than the low-expressed tumor tissues (Fig. **2G**), implying that UBTD1 promotes tumor stromal proliferation and obstructs immune cell infiltration. In addition, we assessed the expression levels of “classic” immune checkpoints in both groups and found that UBTD1 expression was significantly positively correlated with CTLA4 and PD-L1, with correlation coefficients of 0.24 and 0.33, respectively (Fig. **2H**).

### 3.4 Validation of UBTD1 as an Independent Prognostic Factor

**Fig. (3):**
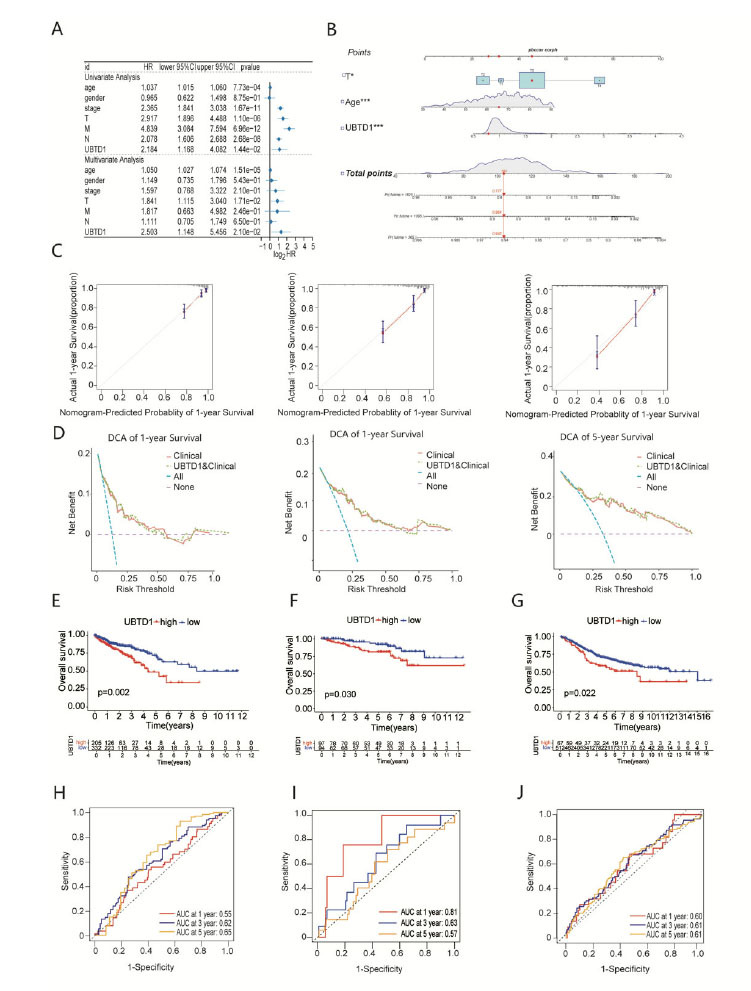
Prognostic signature analysis of UBTD1. (**A**) Forest plot of univariate and multivariate Cox regression analyses with OS in TCGA database. (**B**) At 1, 3, and 5 years, the nomogram predicted survival chances. (**C**) Calibration curves of the nomogram. (**D**) In the TCGA cohort, DCA curves for 1-, 3-, and 5-year survival predictions are shown. (**E-G**) Kaplan-Meier survival curve analysis in the high-UBTD1 and low-UBTD1 patients with CRC in TCGA, GSE87211, and GSE39528 cohorts. (**H-J**) ROC curves evaluating the predictive ability of 1-, 3-, and 5-year survival rates by comparison with the expression of UBTD1.

As high expression of UBTD1 was significantly associated with immune escape in CRC, we screened the clinical characteristics (including age, sex, and stage) of patients in the CRC cohort derived from the TCGA database to determine whether UBTD1 could serve as an independent predictor of prognosis in patients with CRC. Assessment of the independent performance of these clinical features revealed that tumor malignancy (*P* < 0.05, HR = 2.365), age (*P* < 0.05, HR = 1.037), and UBTD1 expression (*P* < 0.05, HR = 2.184) were independent predictors of prognosis in patients with CRC (Fig. **3A**). Consequently, a nomogram was constructed to predict the probability of survival for patients with CRC during 1, 3, and 5 years as independent predictors (Fig. **3B**). To further validate the accuracy of the nomogram, a calibration curve exhibiting a slope of 45° was added (Fig. **3C**), which indicated the reliability of the nomogram predictions. In addition, the Decision Curve Analysis (DCA) curves showed a net benefit of UBTD1 expression in predicting our diagnostic model (Fig. **3D**).

As UBTD1 has been identified as a prognostic factor, we named the TCGA cohort as the practicing set, the GSE 39582 cohort, and the GSE87211 cohort as the validation sets, and then performed survival analysis using the Kaplan-Meier method. Figs. (**3E**-**G**) illustrate that patients with CRC with higher UBTD1 expression had a poorer prognosis.

ROC and Area Under the Curve (AUC) analyses of the TCGA cohort also revealed that UBTD1 could be an independent prognostic feature. The AUC was 0.55 at the end of the first year, 0.62 at the end of the third year, and 0.65 at the end of the fifth year (Fig. **3H**). In the validation group, the AUCs at the end of the first, third, and fifth year were 0.81, 0.63, and 0.57, respectively, as per the GSE87211 cohort (Fig. **3H**). Additionally, the AUCs at the end of the first year were 0.60, at the end of the third year were 0.61, and at the end of the fifth year were 0.61, as per the GSE39582 cohort (Figs. **3I** and **J**). Therefore, UBTD1 may be considered an independent prognostic factor in patients with CRC.

### 3.5 Prediction of Potential Therapeutic Strategies Targeting UBTD1

**Fig. (4):**
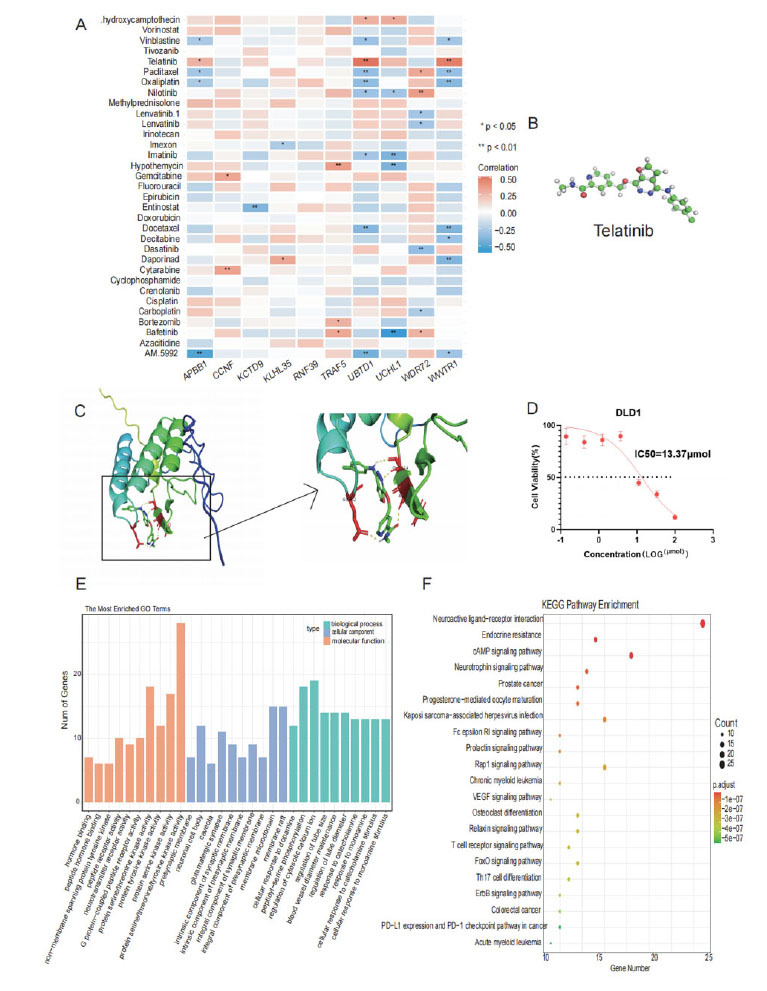
Prediction of therapeutic agents related to UBTD1. (**A**) Correlation between therapeutic compound activities and the expression of DPURGs. Red represents a positive correlation, and blue represents a negative correlation. * *P*< 0.05, ** *P* < 0.01. (**B**) 3D molecular structure of telatinib. (**C**) Diagram of telatinib and protein combination. (**D**) The half-maximal inhibitory concentration of telatinib in DLD1 cells. (**E** and **F**) GO and KEGG analyses of telatinib target genes.

To investigate potential therapeutic strategies targeting ubiquitination-related genes, we obtained the average Z scores of potential drugs associated with RNA expression in CRC from the CellMiner (DTP NCI-60) database. Subsequently, we predicted the efficacy of the 33 drugs and assessed the relationship between drug expression and DPURGs. We found that among the 33 drugs, telatinib had the strongest correlation with UBTD1, indicating that the higher the expression of UBTD1, the greater the sensitivity of telatinib (Fig. **4A**). Telatinib is a selective inhibitor of VEGFR2, VEGFR3, c-Kit, and PDGFRα, the receptor for vascular endothelial growth factor (VEGFR), which plays an essential role in stimulating angiogenesis and vascular regeneration [36]. Fig. (**4B**) shows that the 3D structure of telatinib with GLU at position 62, ARG at position 112, and GLN at position 114 of UBTD1 formed noncovalent bonds with telatinib (Fig. **4C**). The minimum binding energy of UBTD1 to telatinib was -8.72 cal/mol, indicating that telatinib could be used as a potential target drug in patients with high UBTD1 expression. To further verify the effect of telatinib on CRC cells, we assayed the effect of telatinib on DLD1 activity using the CCK8 assay and determined its half-maximal inhibitory concentration (IC_50_) as 13.37 μmol (Fig. **4D**). Subsequently, we obtained the telatinib target genes from the compound target prediction database [37] (https://www.sib.swiss/). Our analyses of these target genes using GO and KEGG pathway analyses revealed that telatinib exerts a significant effect on angiogenesis-related pathways, such as the VEGF signaling pathway, as well as tumor immune-related pathways, including PD-L1 expression and PD-1 checkpoint pathway in cancer, and T cell receptor signaling pathway, and its impact extends beyond CRC to prostate cancer and acute myeloid leukemia (Figs. **4E** and **F**).

### 3.6 Experimental Verification

**Fig. (5):**
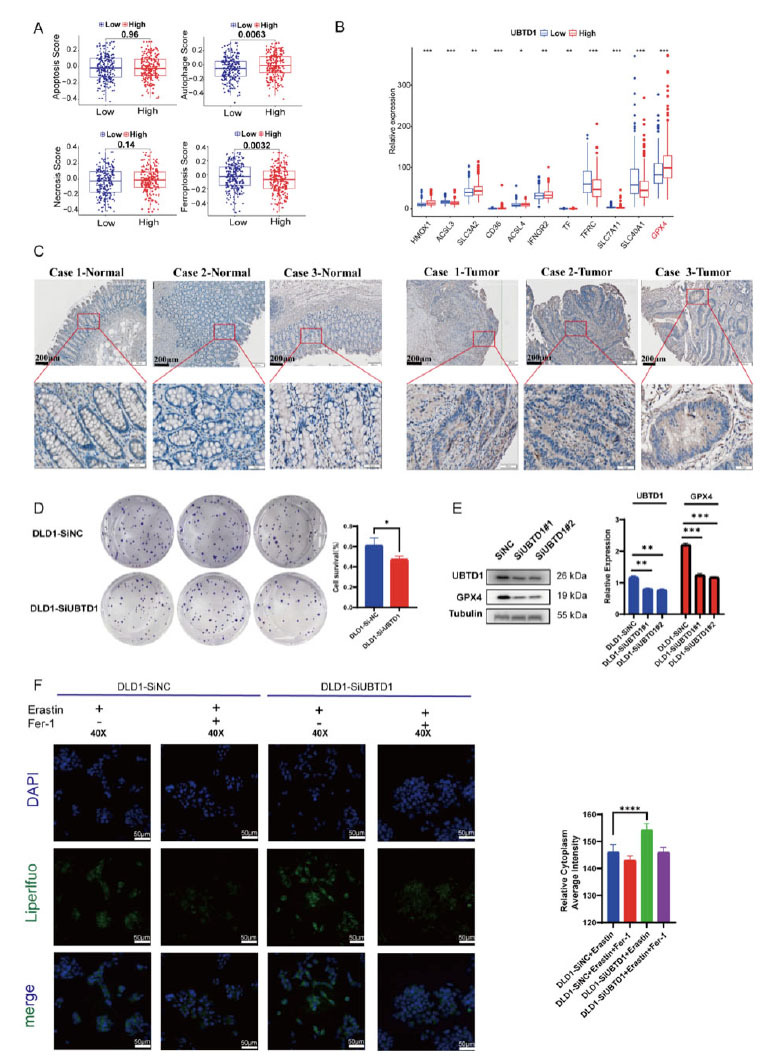
Experimental verification. (**A**) Scores of apoptosis, necrosis, autophagy, and ferroptosis between high- and low-expression of UBTD1. Correlation between UBTD1 expression and apoptosis, necrosis, autophagy, and ferroptosis scores. (**B**) Box plots showing the differences in ferroptosis markers between the two UBTD1 groups. (**C**) Immunohistochemical expression of UBTD1 in paracancerous tissues (40X and 20X magnification). (**D**) Cloning experiments show a reduced proliferative capacity after SiUBTD1 treatment. (**E**) Western blotting showing that GPX4 was significantly reduced after SiUBTD1 treatment. (**F**) Representative immunofluorescence image of lipid peroxidation in DLD1 cells at 40X magnification.

We analyzed the relationship between UBTD1 and various programmed cell death pathways in CRC tissues using a database of apoptosis, necrosis, autophagy, and ferroptosis. The findings demonstrated that UBTD1 had an apparent positive correlation with autophagy and ferroptosis and was independent of apoptosis and necrosis (Fig. **5A**). UBTD1 showed the strongest correlation with ferroptosis. Therefore, we characterized the regulatory role of UBTD1 in ferroptosis-related pathways. (Fig. **5B**) shows the expression of ferroptosis-related proteins in different groups.

To further verify the clinical feasibility of UBTD1 as an independent prognostic factor, immunohistochemical staining of UBTD1 was performed on CRC and adjacent tissues. We demonstrated UBTD1 immunohistochemical staining of three of these pairs of tissues, analyzed the difference in the immunohistochemical expression of UBTD1 between the two groups, and found that UBTD1 was a reliable marker (Fig. **5C**).

We then used siRNA to knock down UBTD1 in DLD1 cells and validated the results of bioinformatics analysis by clone formation and western blotting. The results showed that the UBTD1 knockdown group significantly slowed down the growth of cancer cells (Fig. **5D**). Fig. (**5E**) shows that the critical regulator of ferroptosis, GPX4, was also knocked down in the UBTD1 knockdown group. These results indicate that UBTD1 may act as a suppressor of ferroptosis to inhibit the progression of CRC by mediating immune escape. To further validate the role of UBTD1 in ferroptosis, we examined the lipid peroxide content of DLD1-NC and Si in cells treated with activated or inhibited ferroptosis. These results confirmed that lipid peroxide fluorescence intensity was significantly enhanced after UBTD1 knockdown, indicating that UBTD1 plays an inhibitory role in ferroptosis. Additionally, the addition of Fer-1 inhibited the enhancement of lipid peroxidation induced by UBTD1 knockdown (Fig. **5F**).

## 4 DISCUSSION

Ubiquitination is critical for many physiological processes, including cell survival, differentiation, autophagy, homeostatic regulation, innate immunity, and adaptive immunity [11]. During DNA repair, dysregulation of the ubiquitination system results in the incorrect or inadequate assembly of protein complexes. This may lead to tumorigenesis and abnormal activation or inactivation of cellular metabolic pathways [12, 38]. However, the relationship between ubiquitination, tumorigenesis, and immune function is not fully understood, and identifying immune target sites associated with ubiquitination could facilitate immunotherapy for tumors. Therefore, we screened ubiquitination-associated prognostic genes in CRC and utilized bioinformatics to analyze their association with oncogenic hallmark pathways and their correlation with the immune microenvironment and checkpoints. Based on these predicted genes, we found that UBTD1 is highly immune-related and may be used as a novel target to inhibit tumor immune escape.

Ubiquitin-containing structural domain protein 1 (UBTD1) is a ubiquitin-like protein that regulates E2 ubiquitin transferase and E3 ligase [11, 12]. Although the biological function of UBTD1 remains unclear, it has been reported that UBTD1 regulates cellular aging through a positive feedback loop involving TP53 [18]. As demonstrated in Fig. (**1**), UBTD1 expression is significantly elevated in CRC tissues compared to normal tissues. The survival analysis further corroborates that high UBTD1 expression is associated with poorer overall survival in CRC patients.

During tumorigenesis, tumors evolve to evade the immune system *via* immune checkpoints, such as PD-1 and CTLA4 [39, 40]. These immune checkpoints restrict T cell activation and inhibit immune effects in the immune system. Therefore, we explored the role of UBTD1 in antitumor immunity. The results showed that UBTD1 expression was significantly correlated with immune and stromal and estimated scores and tumor purity in patients with CRC. The UBTD1 high-expression group tended to have higher immune and stromal scores, suggesting that UBTD1-mediated immune escape may be mediated by factors, such as immune checkpoint molecules and proliferation of the tumor stroma. Our data revealed that most clinical immune checkpoints, including CTLA4 and PD-L1, were positively correlated with UBTD1 expression. In particular, high expression of UBTD1 was associated with the overexpression of immune checkpoint molecules, which suppressed the body's immune function, suggesting that UBTD1 may help achieve immune escape through its pro-expression effect on immune checkpoints.

Given the correlation of UBTD1 in clinical prognosis and immune checkpoint expression, it is meaningful to find potential therapeutic drugs for UBTD1. The VEGFR inhibitor tadalatinib has the potential to be a therapeutic agent for CRC patients with high UBTD1 expression. Molecular docking analysis showed that tadalatinib has a strong binding affinity with UBTD1, indicating that the drug may be able to effectively target the UBTD1-mediated pathway in CRC. However, the specific pharmacological effects need to be further explored.

Tumor heterogeneity is characterized by dysregulated cell death, and the dysregulation of lipid metabolism is a hallmark of the tumor microenvironment (TME). Ferroptosis is a form of cell death that is primarily characterized by iron-dependent oxidation of phospholipid membranes that triggers cell death [41]. In this study, we found that UBTD1 influenced ferroptosis in CRC. The correlation between UBTD1 and ferroptosis sensitivity can be explained as follows: First, UBTD1 is a ubiquitination-associated protein whose role is considered in the degradation function; it is now known that UBTD1 acts on UBE2D, which is closely associated with the Fe2+ transport stimulator (SFT), affecting the transport of ferroptosis and eventually regulating the Fenton reaction and thus lipid peroxidation reactions [16]. Second, UBTD1 knockdown decreases the ubiquitination of YAP and leads to the activation of YAP downstream signaling [42, 43]. It has been reported that YAP upregulates several ferroptosis regulators, including ACSL4 and TFRC. Finally, we verified that the knockdown of UBTD1 could affect GPX4, a key protein in iron death, but the mechanism involved needs to be further explored [44]. In summary, UBTD1 may regulate ferroptosis through multiple mechanisms, and further experimental exploration of these mechanisms is warranted.

## STUDY LIMITATIONS

Our study has some limitations. First, information on the tissue samples used for bioinformatics analysis was obtained from the TCGA and Gene Expression Omnibus databases, and retrospective studies were conducted. Furthermore, the correlation between UBTD1 and immune escape was investigated without considering the effects of other factors on immunosuppression. There is no experimental evidence for the involvement of UBTD1 in immune escape and ferroptosis. However, the specific mechanisms through which UBTD1 affects or regulates immune checkpoints and ferroptosis-related proteins remain unclear and require further investigation.

## CONCLUSION

Our study revealed that UBTD1, a novel ubiquitination-associated gene, is strongly associated with prognosis and immunity. In particular, high UBTD1 expression in CRC is associated with a poor prognosis and may promote tumorigenesis and progression by inhibiting ferroptosis. Additionally, UBTD1 expression is significantly associated with the majority of immune checkpoints. In CRC, high UBTD1 expression may help cancer cells evade immunity *via* checkpoints. Therefore, UBTD1 is a potential prognostic marker, regulator of ferroptosis, and modulator of resistance to immune checkpoint blockade therapy.

## Data Availability

The datasets generated and/or analysed during the current study are available in the TCGA repository, [portal.gdc.cancer.gov]; GEO repository, [https://www.ncbi.nlm.nih.gov/geo/query/acc.cgi?acc=GSE39582] and [https://www. ncbi.nlm.nih.gov/geo/query/acc.cgi?acc=GSE87211] and [w ww.ncbi.nlm.nih.gov/geo/query/acc.cgi?acc=GSE188711]. The datasets generated during and/or analysed during the current study are available from the corresponding author upon reasonable request.

## LIST OF ABBREVIATIONS

CNV: Copy Number Variation
CRC: Colorectal Cancer
DPURGs: Differential Prognostic Ubiquitination-related Genes
DUB: Deubiquitinating Enzymes
GDSC: Drug Sensitivity in Cancer
GSEA: Gene Set Enrichment Analysis
GSVA: Gene Set Variation Analysis
KM: Kaplan-Meier
MMR: Mismatch Repair Gene Status
MSI: Microsatellite Instability
OS: Overall Survival
TCGA: The Cancer Genome Atlas
TCR: T Cell Receptor
TIDE: Tumor Immune Dysfunction and Rejection
TMB: High Mutation Burden
TME: The Tumor Microenvironment
UBTD1: Ubiquitin-containing Structural Domain Protein 1
UPS: Ubiquitin-proteasome System
