# Supplementary material for: Ubiquitination-related Gene UBTD1 Mediates Poor Prognosis of Colorectal Cancer and Affects Colorectal Cancer Cell Proliferation and Ferroptosis
Source: Recent Pat Anticancer Drug Discov. 2025 Jan 15;21(2):126–40. doi: 10.2174/0115748928323408241002131753 (PMC13312376; doi:10.2174/0115748928323408241002131753)
Supplement: Supplementary file 1 [file PRA-21-2-126_SD1.pdf]

SUPPLEMENTARY MATERIAL

Ubiquitination-related Gene UBTD1 Mediates Poor Prognosis of Colorectal Cancer and Affects Colorectal Cancer Cell Proliferation and Ferroptosis

Yuzhao Jin[1,2,3,#], Luyu Liao[1,2,3#], Qianping Chen[1,2,3], Bufu Tang[4], Jin Jiang[5], Ji Zhu[1,2,3,*], Minghua Bai[3,*] and Lingjiao Guo[6]

[1]Postgraduate training base Alliance of Wenzhou Medical University (Zhejiang Cancer Hospital), Hangzhou, Zhejiang, 310022, China; [2]Hangzhou Institute of Medicine (HIM), Chinese Academy of Sciences Hangzhou, 310000, China; [3]Department of Abdominal Radiotherapy, Zhejiang Cancer Hospital, Hangzhou, 310000, China; [4]Department of Radiotherapy, Zhongshan Hospital, Shanghai, 200032, China; [5]Department of Radiotherapy, First Hospital of Jiaxing, Jiaxing, 31400, China; [6]Yuhuan Second People's Hospital, Health Community Group of Yuhuan Second People's Hospital, Taizhou, 317605, China

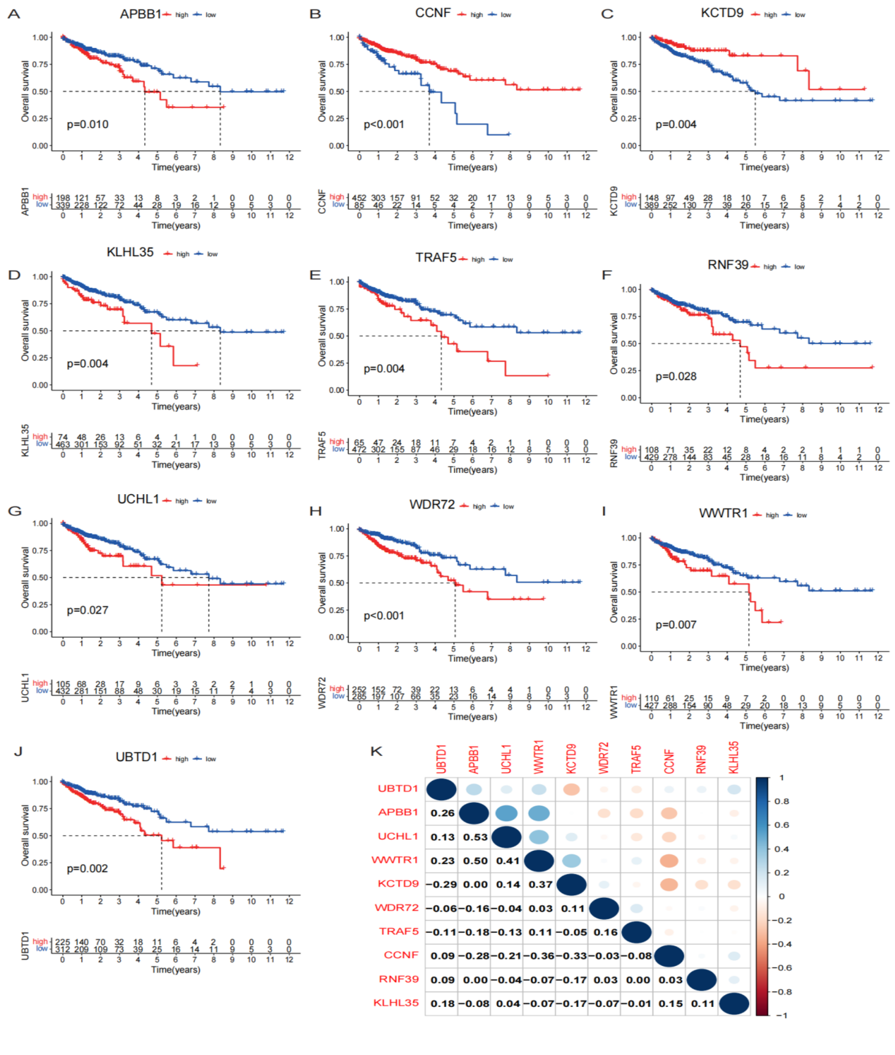

Fig. (S1). Functional enrichment of UBTD1-related DEGs in TCGA-COAD/READ cohort. J) KM curves for the survival analysis of DPURGs. (K) Correlation between gene expression of DPURGs in TCGA cohort.

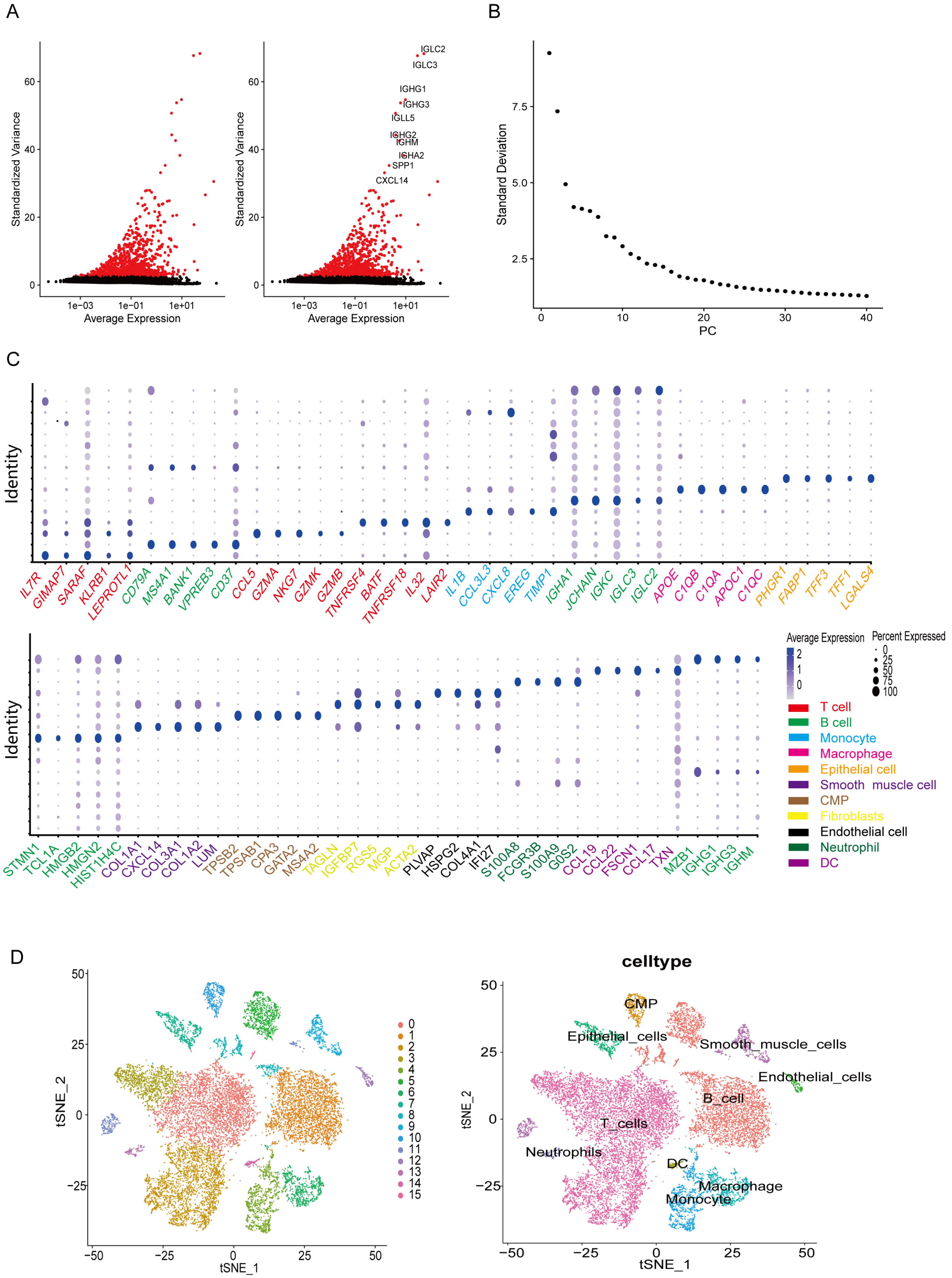

Fig. (S2). Identification of multiple cell types by single-cell multiomics sequencing. (A) The top 2000 highly heterozygous genes were screened. (B) The scree plot shows the best PC number. (C) The dot plot shows the top five different genes in the 15 groups. (**D**) The tSNE of all cells from six patients with CRC. Cells are clustered into ten groups according to known marker genes: T lymphocytes, B lymphocytes, monocytes, macrophages, epithelial cells, smooth muscle cells, CMP, fibroblasts, endothelial cells, neutrophils, and DCs.

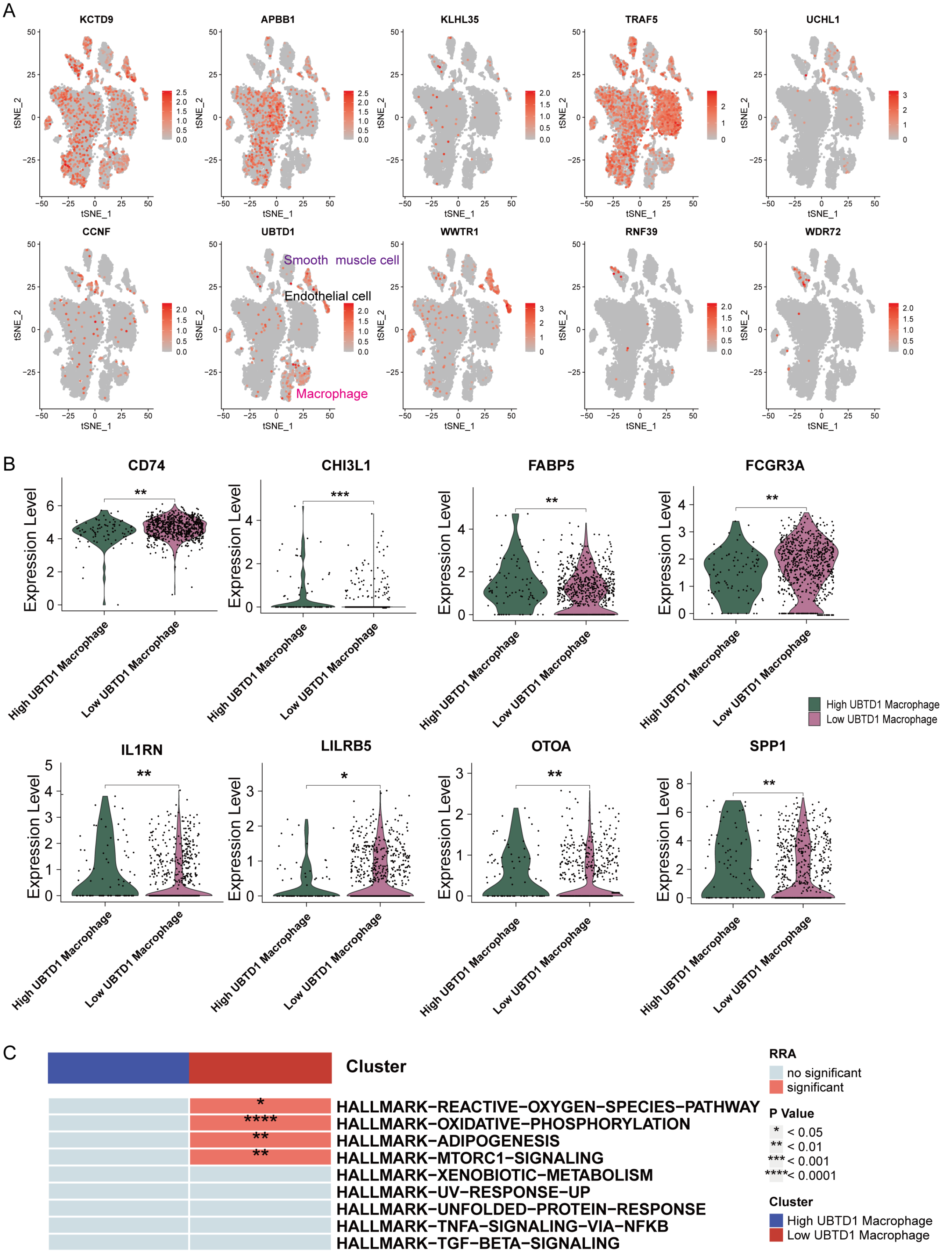

Fig. (S3). Single-cell analysis reveals the correlation between UBTD1 and macrophages. (**A**) DPURGs were identified in the single-cell tumor atlas in the tSNE plots. (**B**) Differential gene expression in macrophages with high and low UBTD1 expressions. (**C**) Top ten differential pathways in macrophages with high and low UBTD1 expressions.
